# Supplementary material for: To call a cloud ‘cirrus’: sound symbolism in names for categories or items
Source: PeerJ. 2017 Jun 29;5:e3466. doi: 10.7717/peerj.3466 (PMC5493972; doi:10.7717/peerj.3466)
Supplement: Supplemental Information 1 — The ratings for the roundness and complexity of the Vincasso stimuli used in this study. [file peerj-05-3466-s001.pdf]

Vinčasso Stimuli: Design and norming

A novel set of pencil-drawn visual stimuli were created for investigations of sound symbolic effects in stimuli with more subtle and complex variation than some of the better known example in the literature on sound symbolism. The twelve items were designed to fall into two general visual categories, on the basis of their rounded versus angular forms. We first describe these stimuli in Ković, Sučević and Styles (under review).

> *Visual stimuli were abstract pencil drawings of novel complex shapes designed for use in a number of different studies. The forms were inspired by the shape of the historic Vinča figures, products of a Neolithic culture from the Balkan region. These abstract drawings were created to form two categories, differing in their visual properties: one category consisted of curved shapes, in rounded forms, and the other category, angular shapes, in a vertical orientation (see Figure 1B). Each category contained six members. Given their abstract forms (reminiscent of Picasso), we term these stimuli Vinčasso illustrations.*

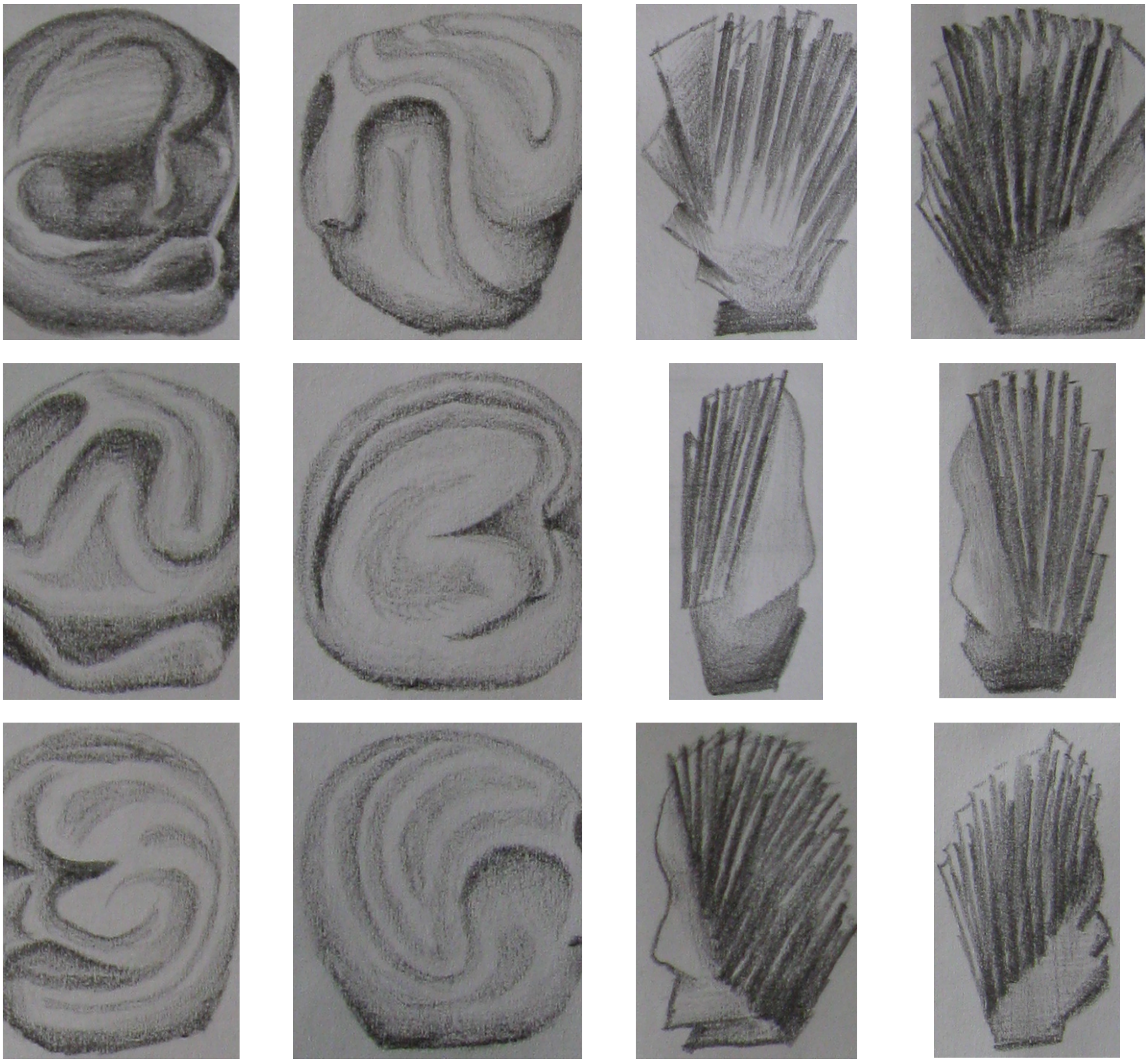

Figure 1. The 12 Vinčasso Stimuli at full size. From Top-to-Bottom, in columns from the left to the right, the stimulus numbers are 11, 12, 13 / 14, 15, 16 / 31, 32, 33 / 34, 35, 36.

In a rating study, we asked 135 Undergraduates at a Serbian University to rate each of these visual stimuli on a scale from 1 (angular) to 7 (round), using the online Qualtrics survey platform. Stimuli were presented in a random order. Participants were also asked to rate each stimulus for its Visual complexity on a seven point scale from 1 (simple) to 7 (complex). The figures are given overleaf, in Figure 1. The digital stimuli used in the main experiment can be found in the Open Science Framework repository for this article (link here).

The raw rating responses are given in the Open Science Framework repository for this article (link here). Mean and median ratings for each stimulus items are given in Table 1. It is clear that the range of rating for each stimulus varies across the full scale, however, median responses are clearly clustered at the ends of the scale for the rating of Angularity/Roundness.

Table 1. Rating Data for the Vinčasso Stimuli.

Group *	Shape-type	Stim Number	Angular/ Round			Simple/ Complex		
			Median	Range	Mean	Median	Range	Mean
0	round/soft	11	1	1-5	1.5	6	1-7	5.5
0	round/soft	12	1	1-7	1.6	5	1-7	4.9
0	round/soft	13	1	1-7	1.5	4	1-7	4.3
0	round/soft	14	1	1-7	1.8	4	1-7	4.3
0	round/soft	15	1	1-7	1.6	3	1-7	3.5
0	round/soft	16	1	1-7	1.5	3	1-7	3.4
1	angular/hard	31	7	1-7	6.3	4	1-7	4.1
1	angular/hard	32	5	1-7	5.1	3	1-7	3.4
1	angular/hard	33	6	1-7	5.8	4	1-7	4.2
1	angular/hard	34	6	1-7	5.7	5	1-7	4.7
1	angular/hard	35	6	1-7	5.4	3	1-7	3.6
1	angular/hard	36	6	1-7	5.9	4	1-7	3.6

* 0 refers to the ‘round’ category of objects, 1, the ‘angular’ category

** Prefix digit indicates which visual object group the picture belongs to: 1=Group 0, 3=Group 1

Curved/Angular Ratings

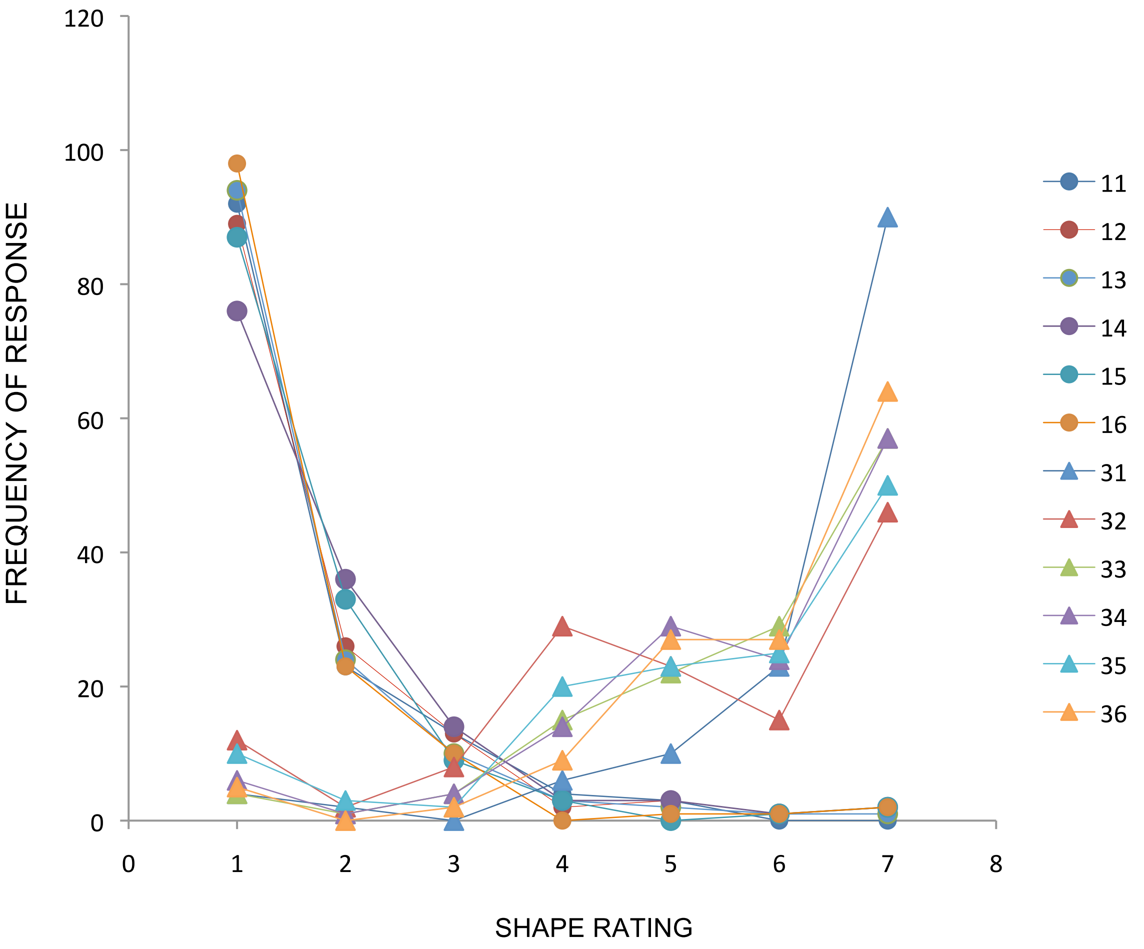

Fig 2. Frequency of response for rating of Angularity/Roundness for the 12 visual stimuli. Stimulus numbers beginning with 1 were designed to belong to the 'round/soft' category. Stimulus numbers beginning with 3 were designed to belong the 'angular/hard' category.

Figure 2 gives the frequency with which people gave different ratings of Angularity/Roundness for each stimulus, where it is clear that there are two clusters of values for stimuli designed to fall into the two visual categories: The first six stimuli attract a large number of '1' ratings (indicating angularity), and the second six stimuli attract a large number of '7' ratings (indicating roundness). A Mann Witney U test on the raw individual ratings confirms that the response patterns for stimuli in the two shape groups are indeed significantly different ($Z(1614)=32.2$, $p<.0005$), as does an independent samples t-test on the mean rating values for each item (Round: $M = 1.58$, $SD = .10$; Sharp: $M = 5.68$, $SD = 0.43$; $t(5.51) = 22.98$, $p < .001$).

Complexity Ratings

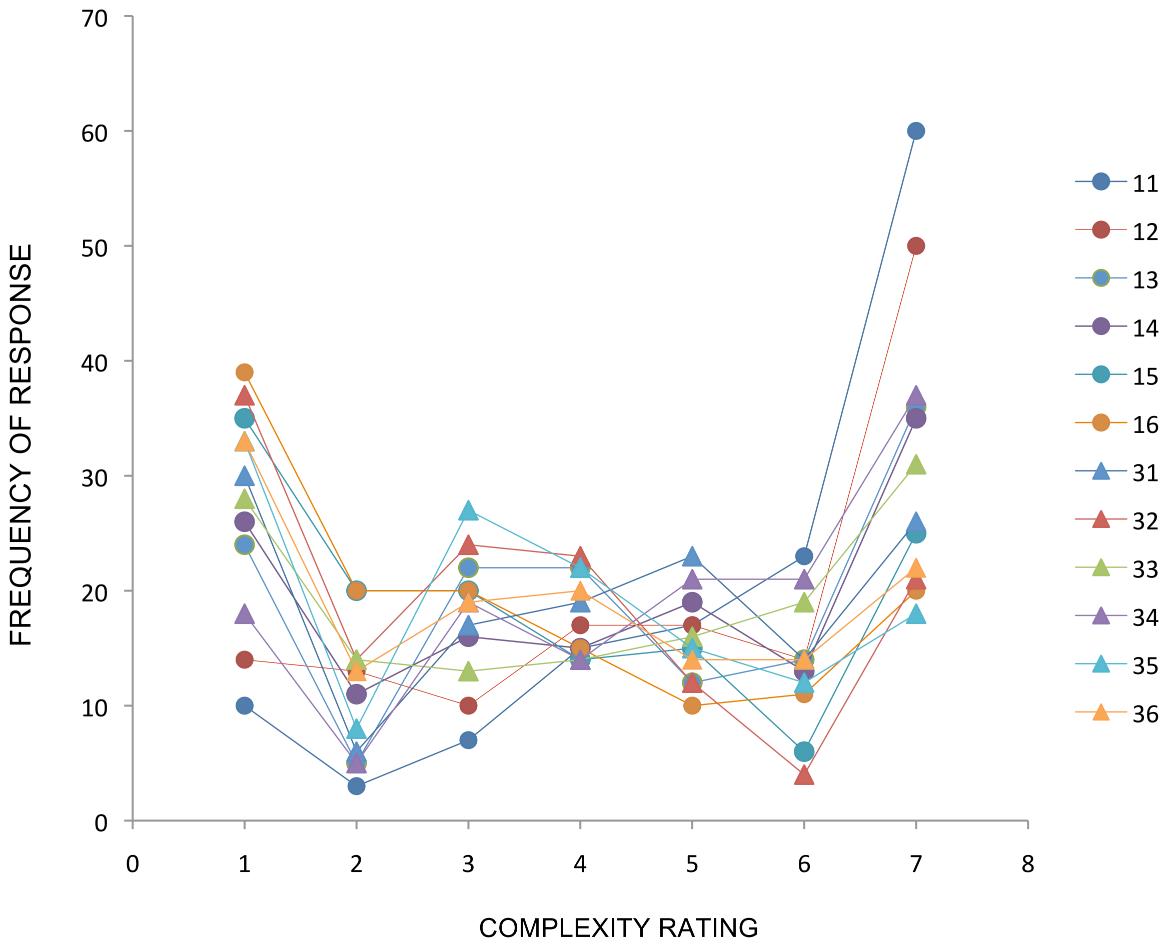

Fig 3. Frequency of response for ratings of Complexity for the 12 visual stimuli. Stimulus numbers beginning with 1 were designed to belong to the 'round/soft' category. Stimulus numbers beginning with 3 were designed to belong the 'angular/hard' category.

Figure 3 gives the frequency with which people gave different ratings of Complexity for each stimulus, where there is no clear-cut distinction between stimuli designed to fall into the two visual categories. Although a Mann Witney U test on the raw individual ratings suggests there may be a subtle difference in the relative rankings of the stimuli from two visual categories ($Z(1620)=3.4$, $p=.001$), as expected, this pattern is not borne out when participant-by-item variability is controlled by evaluating differences between the mean rating values for each item (Round: $M = 4.30$, $SD = .80$; Sharp: $M = 3.95$, $SD = .45$; $t(10) = .954$, $p = .37$).

We therefore conclude that the Vinčasso Stimuli differ reliably in the expected visual dimension (Angularity/Curvature), but do not differ reliably in another dimension known to influence object processing (Complexity).
