# Supplementary material for: To call a cloud ‘cirrus’: sound symbolism in names for categories or items
Source: PeerJ. 2017 Jun 29;5:e3466. doi: 10.7717/peerj.3466 (PMC5493972; doi:10.7717/peerj.3466)
Supplement: Supplemental Information 2 — The analysis of pitch and intensity of pseudowords used in the study previously validated for their soft/sharp associations. [file peerj-05-3466-s002.pdf]

Auditory Stimuli: Acoustic Profiles

12 Pseudowords previously validated for their soft/sharp associations were used in the main study. As prosody is understood to contribute to the constellation of acoustic cues which can drive sounds symbolism (Dingemanse, Schuerman, Reinisch, Tufvesson, & Mitterer, 2016; Nygaard, Cook, & Namy, 2009), we offer visualizations of the prosodic characteristics of the 12 auditory stimuli used in the current study.

Condition*	Word	IPA	filename
0	bimun	/bimun/	bimun.wav
0	lavon	/lavon/	lavon.wav
0	koluv	/koluv/	koluv.wav
0	munel	/munel/	munel.wav
0	vadem	/vadem/	vadem.wav
0	volab	/volab/	volab.wav
1	čičaš	/ʈʂiʈʂaʂ/	cicas.wav
1	kažanj	/kaʒaɲ/	kazanj.wav
1	rarač	/ɾaɾaʈʂ/	rarac.wav
1	šičak	/ʂiʈʂak/	sicak.wav
1	torar	/toɾaɾ/	torar.wav
1	zukač	/zukaʈʂ/	zukac.wav

*0 here *round*, and 1 denotes *angular* sounding pseudowords. The creation of these words is described in detail elsewhere (Sučević, Savić, Popović, Styles, & Ković, 2015).

Pitch and Intensity were computed in PRAAT (Boersma & Weenink, 2013). Insensity was computed using standard settings. To reduce inaccurate fundamental frequency mapping during voiceless segments, audio were filtered to remove values below 500Hz before Pitch was tracked. Pitch tracks were manually corrected to remove frequency doubling, and other perceptual artefacts. Figure 1 shows the resulting Pitch tracks for all audio stimuli.

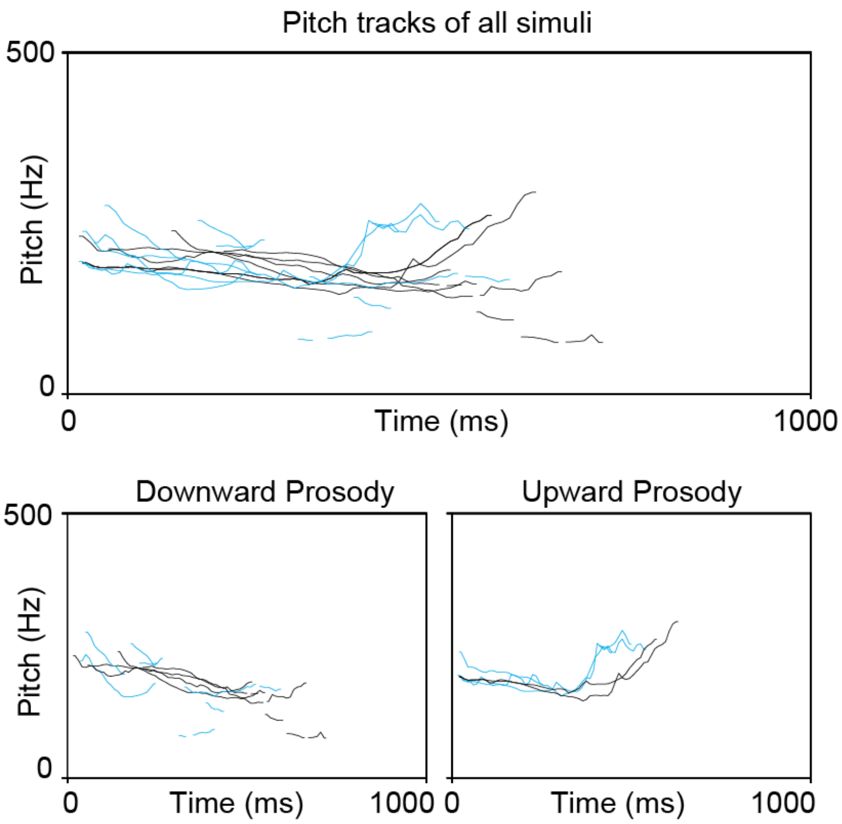

Figure 1. Pitch tracks of audio files used as stimuli in the main experiment. TOP. All stimuli overlayed, with stimuli known to be matched with *rounded shapes* in BLACK, and stimuli known to be matched with *angular shapes* in BLUE. BOTTOM. Pitch tracks for stimuli with predominantly upward or downward inflection over the two syllables of the pseudoword.

From the pitch tracks in Figure 1 (Top), it is clear that audio labels for the two types of stimuli shared a similar range of picth values, with highest, lowest and steady state values (median pitch values) substantially overlapping for stimuli of the two kinds. The pitch tracks for *angular-sounding* pseudowords tend to be shorter than the tracks for *round-sounding* pseudowords, and this can be understood as an outcome of the voiceless nature of the final consonant in the majority of the angular sounding pseudowords. Two of the angular-sounding pseudowords have an upwards inflection with a sustained peak value at the end of the utterance. These two stimuli end with the Serbian inter-dental retroflex tap, so the sustained high pitch portion is a necessary outcome of the articulation of this segment.

Figure 2 shows the Intensity of all stimuli, where it is clear that intensity profiles in the bisyllabic stimuli are quite variable, and substantially overlap between angular-sounding and round-sounding pseudowords.

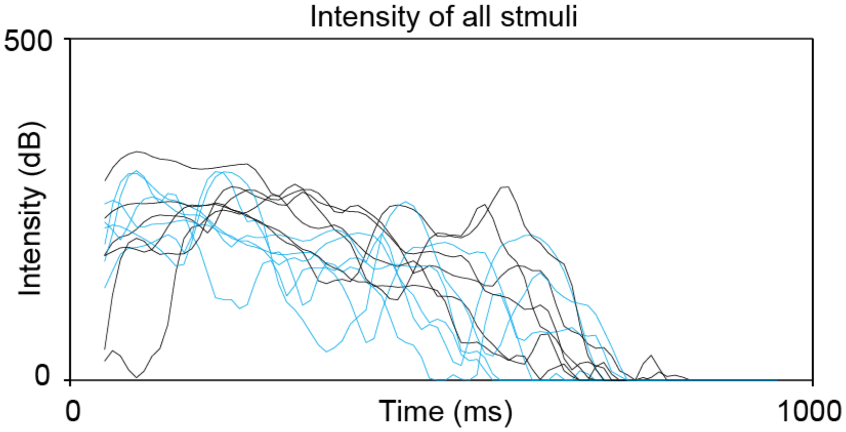

Figure 2. Intensity of audio files used as stimuli in the main experiment. TOP. All stimuli overlayed, with stimuli known to be matched with *rounded shapes* in BLACK, and stimuli known to be matched with *angular shapes* in BLUE. BOTTOM. Pitch tracks for stimuli with predominantly upward or downward inflection over the two syllables of the pseudoword.

References

Boersma, P., & Weenink, D. (2013). *Praat: doing phonetics by computer*.

Dingemanse, M., Schuerman, W., Reinisch, E., Tufvesson, S., & Mitterer, H. (2016). What sound sybolism can and cannot do: Testing the iconicity of ideophones from five langauges. *Language, 92*(2), e117-e133.

Nygaard, L. C., Cook, A. E., & Namy, L. L. (2009). Sound to meaning correspondences facilitate word learning. *Cognition, 112*, 181-186.

Sučević, J., Savić, A. M., Popović, M. B., Styles, S. J., & Ković, V. (2015). Balloons and bavoons vs spikes and shikes: ERPs reveal shared neural processes for shape-sound-meaning congruence in words, and shape-sound congruence in pseudowords. *Brain & Language, 145-146*, 11-22. doi: http://dx.doi.org/10.1016/j.bandl.2015.03.011
