# Supplementary material for: To call a cloud ‘cirrus’: sound symbolism in names for categories or items
Source: PeerJ. 2017 Jun 29;5:e3466. doi: 10.7717/peerj.3466 (PMC5493972; doi:10.7717/peerj.3466)
Supplement: Supplemental Information 3 — Misinterpreting the purpose of the task by participants and our approach to it. [file peerj-05-3466-s003.pdf]

Experimental Pragmatics and the Experimenter-As-Idiot Fallacy
Or
The Curious Case of the Poorly Understood Instructions

A mystery in data, solved by

Suzy J Styles[1]
&
Vanja Kovic[2]

Summary

We report a case where experimental instructions were misunderstood by approximately two-thirds of the participants in an experiment. By (mis)interpreting the purpose of the task, they assumed the wording of our instruction text had contained an error (*The Experimenter-as-idiot Fallacy*): They answered the question *they thought we intended to ask*. We diagnose that participants were guided by how the experiment was structured, and discuss this in terms of Experimental Pragmatics. We conclude with guidelines and suggestions on how to avoid similar traps in future work. This Case Report may find further use in the context of training junior researchers in Laboratory procedures, or in general Research Methods training.

This report appears in the Supplementary Materials of Kovic, Sucevic & Styles (under Review), *PeerJ*.

[1]Department of Psychology, University of Belgrade, Belgrade, Serbia
[2]Division of Psychology, Nanyang Technological University, Singapore

The Mystery

In 2014, we conducted two experiments, where the results made no sense. Task compliance was high, and accuracy was good in all but one set of test trials. In that set of trials, error rates were off the charts, and the remaining data did not form a coherent pattern. How could we explain the discrepancy? And what should we do with the data?

The Task

In the experiments, participants went through two blocks of training, followed by a test. In the test, participants were asked to decide whether two pictures were "identical." The pictures were presented on a computer screen at the same time, side-by-side. There were three types of trials in the test. Identity-Match trials, where people saw exactly the same picture, Category Match Trials, where people saw two pictures drawn from same category (items with similar visual features), and Mismatch Trials, where people saw two pictures drawn from different categories (for full methods, see main article).

Decisions about whether two images are "identical" can be performed without any prior knowledge. However, we were investigating whether some form of training might influence the speed of identity judgments by changing the way people paid attention to objects, thereby influencing recognition processes. For training, people simply had to learn by guessing which object belonged to one of two categories. The training involved seeing each of the test pictures accompanied by an auditory label. They guessed which category it might belong to, and were told whether their guess was "correct" or "incorrect". All of the pictures and the words were novel, and unfamiliar to the participants. The task was conducted in Serbian, the main language of instruction at the University of Belgrade, where the experiments were conducted.

One important feature to note: In the training blocks, participants used keys C and N to guess which category a picture belonged to. In the test block, participants used the same 'C' and 'N' keys to judge whether the pictures were "identical". The allocation of keys was counterbalanced between participants, and clearly laid out in the onscreen instructions at the start of each block. However, it is important to note that, since the function of the keys changed from 'type A/type B' to 'yes/no', it is possible that confusions may arise.

When we analysed the data using a standard procedure, they initially made little sense, as error rates in the Category Match trials were astonishingly high, and analysis of the correct trials resulted in largely unsystematic patterns. This was surprising, as people's overall accuracy was high, and the task didn't seem difficult. After at least a year 'on the shelf' we returned to the data and eventually spotted the source of the problem: There were two distinctly different patterns of responses.

The Data

In general, people were good at figuring out which items were from which categories by the end of the guessing task (the training), with error rates dropping to less than 20% for almost everyone in the second training block. This meant people paid attention to the task, and were following the instructions. Because of participants' typically low error rates, it was possible to allocate the vast majority of participants to a 'response type', by identifying whether their error rates in the test were typically 'low' or 'high' for each of the three types of picture-question. Since the majority of error rates were either very low (0-15%), or very high (85-100%), this was a straightforward process for almost all participants (Although for some participants, the error rate approached 50% in the Category Match condition, meaning that the evaluation may be imprecise for a small number of participants). The spread of these different patterns across conditions is shown in the table below, where the dominant pattern for each group of participants is highlighted.

		% Error for three Test Trial Types: ID Match - Category Match - Mismatch		
		low-low-low	low-high-low	Total
Exp.	Training	A	B	
1. Training w/ Item Labels	Congruent	3	18	21
	Incongruent	12	7	19
2. Training w/ Category Labels	Congruent	7	14	21
	Incongruent	6	15	21
	TOTAL	28	54	82
	Pattern	Expected	Unexpected	
	Interpretation	'identical'	'same category'	

Different participants had answered the questions differently. Some people had interpreted the test question in the way we intended, "Are these pictures Identical? Y/N" (meaning they pressed 'Yes' for Identity Match, and 'No' for the other two conditions), while other people interpreted the question as "According to the training you just did, are these pictures from the same category? Y/N" (meaning they pressed 'Yes' for both Identity Match and Category Match, and 'No' for mismatch).

After finding this anomaly, we double-checked the wording of the original question with a

number of speakers of Serbian to check the logic and interpretation. Everyone we asked agreed with our (standard) interpretation of the wording: "identical" means "identical". And yet, in the context of the experiment, it was clear that different people were interpreting the instructions in different ways. The majority of participants elected the 'same category' interpretation. How could we account for this peculiar interpretation?

Theory of Mind and Experimental Pragmatics

One way of understanding this curious pattern is to realize that participants are trying to make sense of the experiment, while they are performing it. In a kind of disembodied Theory of Mind task, they are trying to figure out what the experimenter *wants them to do*, separately from what the experimenter *asked them to do*. In our experiment, the structure of the training made people pay attention to the categories that pictures belonged to. They therefore assumed that the test would be about the categories they had just learned. We think of this as a form of Gricean Implicature – We would not have given them training about category structure if we did not intend to test it. That is to say, the *pragmatics of the experiment* guided participants to expect a category structure test. Participants could readily dismiss the precise wording of the question as an error on the part of the Experimenter, due to what we call the *Experimenter-as-Idiot* Fallacy.

In fact, we wanted to show that people could tell the difference between different members of the same category, and find out whether their speed would be enhanced/inhibited by the different training regimes. So our wording was actually correct – there was no ambiguity in the text. Indeed, we were pleased to notice that around a third of people did in fact follow the precise instruction, demonstrating that our intended meaning was understandable (if only to some). That said, since the instruction did not match the pragmatics of the task, many of our participants decided for themselves what our intended meaning was.

It should be noted that around two thirds of our participants adopted the Fallacy, although the distribution across training regimes was not uniform. The Fallacy was dominant a) when the training involved just two auditory labels which labelled the categories that were being learned (i.e., Experiment 2. Training with Category Labels), and b) when the training involved different labels for each item in a category, and each label sound was symbolically congruent with the visual properties of the category (Experiment 1. Training with Item

Labels: Congruent). Intriguingly, people were somewhat more protected from the Fallacy when the individual item labels during training were incongruent with the pictures they were paired with (Experiment 1. Training with Items Labels: Incongruent), suggesting that the stimulus combinations may have played a role in participants' interpretation of the task. What should we make of this irregular pattern?

Data Decisions

It is interesting to note that the pattern of errors is somewhat in line with our original predictions – having trained with category labels makes members of a single category seem more similar to each other than having trained with individual item labels (or, by extension, to assume the question is about category structure), and that in the domain of item labels, sound symbolically incongruent labels might help to highlight differences between items, rather than similarities (or, by extension, to assume the question is about individual items, rather than categories).

How to handle the data… Our options include:

a) Analyse the data with 'response pattern' as an additional independent variable
b) Exclude data from participants who used the less-common response
c) Recode 'incorrect' answers as 'correct' according to dominant response type
d) Exclude the problematic condition from further analyses
e) Exclude the condition, but also present the data on distributions of response-type

The implications of each option, and our eventual decision are dealt with in detail below.

Adding the participants' response pattern would be problematic for two reasons. Firstly, as the distribution across conditions is uneven, where the differences between conditions are observed, analysis of subsets may not have the power to show systematic patterns. Perhaps more importantly, since this variable was not included in the study design, including it in the analysis would represent a trip into the Garden of Forking Paths. That is to say, how we chose to implement the analysis would increase the Experimenter Degrees of freedom, and undermine the statistical validity of the work presented in the main article.

Exclusions are often used in Experimental methodologies, especially when it is discovered that some (small) subset of participants have done something other than what the researchers intended. To give a simple example, in paid experiments, sometimes a participant will

quickly press one key repeatedly throughout the experiment, regardless of the specific instructions. Exclusion is a clean, efficient way of dealing with mismatches between the stated instructions and human subjects' ability to follow them. In our case, removing data from all participants who used the minority interpretation would have meant loss of one third of the original participants, and resulted in a substantial loss of power. Furthermore, because of the uneven distribution between conditions, it would have been necessary to run additional participants in Experiment 1 to make up the numbers in the Incongruent training condition). In running those participants, to ensure that they elected the same interpretation as the majority in the other conditions, it would be necessary to change the instructions. Altering the instructions may have further (unexpected) effects on task performance. Hence, when considering loss of power, and possibility of additional complications with instructions, we did not think this was the best approach.

An alternative would be to recode 'incorrect' responses as 'correct' according to each participant's dominant response pattern. This approach would remove the need to exclude participants or conditions. However, this would mean that some participants would be answering 'yes' correctly, and others answering 'no' correctly in this condition, meaning that they would be making their decisions in this condition by pressing different keys. This is problematic as the 'yes' key is the same as the correct response for the Identity Match condition, and the 'no' key is the same as the 'Mismatch' condition. Keys assignments were counterbalanced across participants, so that the number of people with 'yes' as a right-hand response was the same as the number of people with 'yes' as a left-hand response, to account for potential differences in handed response speeds. However, if key-allocations are flipped for a subset of participants in one condition, this may introduce inadvertent response time irregularities, which may be unevenly spread across conditions. In addition, in a small number of participants, error rates were close to 50%, meaning that the allocation of a participant to a response type may have been inaccurate.

To avoid these problems, we elected to remove the problematic condition from all further analysis. Note that in the other conditions, the question interpretation does not matter, as both interpretations give rise to an unequivocal 'yes' for the Identity Match and 'no' for the Category Mismatch trials. We therefore elected to continue with the analysis of the data for those two conditions, without further interference, and to exclude the problematic test trials for all participants. The full analysis of the data can be seen in the main paper.

One additional possibility would be to flag in the main article how the distribution of the Experimenter-as-Idiot Fallacy is in line with the main predictions of the paper, and include statistical analysis of the distributions in the main paper. While this approach has merits, since the dependent variable was not predicted, it could arise out of simple differences in the detail-oriented attention of individuals in different groups. Furthermore, if we did decide to present this as a significant result in the main article, it would represent Post-Hoc Hypothesis, rather than a test of an existing hypothesis, which would undermine the credibility of the research programme. We therefore don't intend to pursue this line of reasoning further (hence the lack of theoretical discussion in the current document), and have not included these data in the main article. We have instead relegated the data, and discussions of its validity to this Supplementary document, with the hope that it serves as an illustrative example.

Conclusions and practical advice

With all of the humility that comes from having made mistakes ourselves, we therefore make the following proposal… People designing experimental research need to remind themselves that their well-thought-out experimental instructions can be ignored *or even overruled* by participants, who may engage in complex Theory-of-Mind interpretations of your experiment. Participants may be trying to guess what you want them to do, and may be subject to confirmation bias, when they see broadly similar instructions. When it comes to rethinking the purpose of the experiment, or rethinking the meaning of a particular word, participants may be more willing to assume you have made a small mistake in your language, than to assume you have made a large error in your logic, as perceived within the context of the experiment. To avoid such pragmatic (mis)interpretations, you need to perform your own theory-of-mind exercise by trying to experience what your experiment would be like from a participant's perspective. This includes conducting several runs of the *full* experiment on yourself… and probably your lab-mates as well.

Suggestions for avoiding similar problems…

1. Try to think through your participants' expectations about what the experiment is for, and why they have to do the different elements of your task.
    a. Is there *ambiguity* in the wording of the question?

b. Is there an *alternative way to interpret* the question (even if unambiguous)?
c. Is there *a simpler question* they might think you want them to answer?
d. Have you provided enough *'scaffolding'* for people to understand what different stages of the task involve?
e. In what ways might people assume *YOU have made a mistake?*

2. Pilot test the logic of your questions on naive participants before beginning an experimental run. This must be done in the context of the experiment, with the usual timing, and cognitive load. The question 'Does that make sense?' should be asked a lot, as well as 'What do you think it means?' even for text that is seemingly obvious.
    a. Consider including *onscreen examples* of any decisions people will make.
    b. Consider including *feedback* throughout the task if possible.
    c. Consider including brief *warmup-blocks* with feedback, for paradigms which won't include feedback.

3. Participants may be paying only a small amount of attention to onscreen instructions, and they may skip/skim text if they think they have understood the 'general gist' of what is included, even though *you need them to pay attention to the details*. Think about how you organize onscreen instructions and how you highlight important features of the task to make the information stick.
    a. Use the *visual layout* of words to highlight contrasts.
    b. Consider about using *colour* to make certain key words 'pop out'
    c. Break up complex instructions into several 'pages', so there is time for retention of each, and to avoid skimming over parts.
    d. *Summarise or recap* multi-part instructions before beginning.
    e. To maximise recall of experimental details, consider *repeating key points*.
